# Admission Braden Scale Score Is Associated with In-Hospital Mortality in ICU Patients Admitted After a Same-Level Fall: A Retrospective Cohort Study

**DOI:** 10.3390/diagnostics16152309

**Published:** 2026-07-23

**Authors:** Temel Kayan, Hasan Oktay Emir, Fatih Seğmen, Hayriye Cankar Dal, Firdevs Tuğba Bozkurt Biçer, Esra Yakışık Aktekin, Cihangir Doğu

**Affiliations:** 1 Department of Intensive Care, Şanlıurfa Mehmet Akif İnan Research and Training Hospital, 63300 Sanliurfa, Türkiye; temel_kayan@hotmail.com; 2 Department of General Intensive Care, Sancaktepe Training and Research Hospital, 34784 Istanbul, Türkiye; hoktayemir@gmail.com; 3 Department of General Intensive Care, Ankara Bilkent City Hospital, 06800 Ankara, Türkiye; drsegmen@gmail.com (F.S.); hayriyecankar@hotmail.com (H.C.D.); firdevstugba.bozkurt@sbu.edu.tr (F.T.B.B.); pavulonmouse@hotmail.com (E.Y.A.)

**Keywords:** Braden scale, ground-level fall, same-level fall, ICU, in-hospital mortality, frailty, risk stratification, geriatric trauma, clinical risk score, older adults

## Abstract

**Background/Objectives:** Ground-level falls (GLFs) are the leading cause of injury-related deaths in older adults, yet a validated bedside tool for early mortality risk stratification remains unavailable. The Braden scale, recorded routinely at intensive care unit (ICU) admission, has shown prognostic value across several critical illness populations. This study aimed to determine whether the admission Braden score is associated with in-hospital mortality in ICU patients admitted following a GLF, and to derive a simple bedside clinical risk score. **Methods:** A single-centre retrospective cohort study was conducted in a 216-bed mixed ICU over a four-year period (January 2020–June 2024). Adult patients admitted to the ICU following a GLF occurring outside the hospital were included. The primary outcome was in-hospital mortality. Multivariable logistic regression was used, and a five-component integer-weighted bedside risk score was derived. Internal validation was performed by 1000-fold bootstrap resampling. **Results:** Among 500 patients (median age 83 years; 63.3% female), 84 (16.8%) died. The admission Braden score was significantly lower in non-survivors (median 12 vs. 13; *p* < 0.001) and was an independent predictor of mortality after multivariable adjustment (adjusted OR 0.821 per one-point increase; 95% CI 0.706–0.954; *p* = 0.010). A five-component bedside risk score—incorporating the Braden score, INR, emergency admission, serum sodium, and procalcitonin—achieved a bootstrap-corrected AUC of 0.833 (95% CI 0.780–0.881) and stratified patients across a 16-fold mortality gradient (5.0% to 79.4%). **Conclusions:** The admission Braden score is associated with in-hospital mortality. When combined with four routine variables, discriminative performance improved. This score has undergone internal validation only. External validation in multicentre prospective cohorts is warranted before clinical use.

## 1. Introduction

Falls from the same level, also known as ground-level falls (GLFs), account for a significant proportion of trauma-related hospital admissions among adults aged 65 and over [1]. They are the leading cause of injury-related deaths in this group. These are classified as low-energy incidents and are associated with high morbidity and mortality in elderly patients. In-hospital mortality ranges from 4 to 18 per cent, whilst one-year mortality can reach 25 per cent [2,3]. Admission to the intensive care unit (ICU) is often required due to traumatic brain injury, haemodynamic instability, the use of anticoagulants, or the presence of multiple comorbidities [4,5]. Unlike the young trauma population, the risk of mortality following GLF is determined not by high-energy trauma but by a reduction in pre-existing physiological reserve [6,7]. Therefore, risk stratification performed at the bedside upon admission to the intensive care unit will enable the determination of treatment goals, the type of monitoring, and the optimisation of resource utilisation in this patient group.

The global burden of fall-related injury in older adults continues to grow. The Global Burden of Disease 2021 study estimated 2.537 million new fall-related hip fractures in China alone in a single year, with a 386% rise in absolute case numbers since 1990 [8]. Fall-related disability also carries a substantial and rising burden among older women, with disability-adjusted life-years attributable to low bone mineral density nearly doubling between 1990 and 2021 [9]. This burden is particularly poorly characterised in rapidly ageing regions such as the Middle East and North Africa, where a recent evidence gap map identified substantial underrepresentation of rigorous fall-outcome research despite a growing at-risk population [10].

The Braden scale, developed in 1987 to predict the risk of pressure injuries, is recorded by nurses upon admission to intensive care units. The scoring system, which assesses six key areas (sensory perception, moisture, activity, mobility, nutrition and friction/shear), is also useful in identifying frailty [11]. Recent studies have extended the prognostic role of this indicator far beyond pressure injuries. A low Braden score has been independently associated with short-term mortality in cardiac intensive care patients [12], patients with traumatic brain injury [13], patients with ischaemic stroke [14], patients with non-traumatic subarachnoid haemorrhage, [15] patients with sepsis [16], patients with dementia [17], and in patients with multiple organ dysfunction syndrome [18]. However, we are not aware of prior studies examining the use of the bedside Braden score for predicting mortality or morbidity following a fall from the same level. In this patient group, frailty may predict patient outcomes. A frailty indicator that can be routinely used at the bedside could significantly improve risk prediction.

The hypothesis of this study was that the Braden scale score recorded upon admission to the intensive care unit (ICU) could be used to predict in-hospital mortality in patients admitted to the ICU following a fall at ground level. A secondary objective was to derive a simple clinical risk score that could be applied at the bedside by combining routine laboratory tests with the Braden score in this population.

## 2. Materials and Methods

### 2.1. Study Design and Setting

This study was conducted at Ankara Bilkent City Hospital. It was carried out retrospectively in the hospital’s 216-bed mixed-type intensive care unit. The study period spanned from January 2020 to June 2024. The study was reported in accordance with the STROBE guidelines for observational studies.

### 2.2. Study Population

Adult patients (aged ≥ 18 years) admitted to the intensive care unit (ICU) from the emergency department, a ward or the operating theatre with a diagnosis of a same-level fall (fall to ground level) occurring outside the hospital. The diagnosis of a same-level fall was confirmed by the initial admission record, the emergency department triage notes and the trauma history taken at presentation. Falls from height, motor vehicle accidents or other traumas were excluded from the study. During the study period, all COVID-19 patients were treated in a separate, dedicated ICU. This was regardless of admission diagnosis. All patients in this analysis were non-COVID cases. Of the 526 patients, 26 with incomplete medical records were excluded, resulting in a study population of 500 patients.

### 2.3. Variables and Outcome Measures

The primary outcome was in-hospital mortality, regardless of cause or timing. The primary predictor was the bedside Braden scale score at the time of admission to the intensive care unit, as assessed during routine nursing rounds. The Braden scale consists of six subscales relating to sensory perception, moisture, activity, mobility, nutrition and friction/shear; the first five subscales are scored from 1 to 4, whilst the friction/shear subscale is scored from 1 to 3, and the subscale scores are summed to yield a total score ranging from 6 to 23. A low total score indicates a greater impairment of physiological reserve.

Variables recorded upon admission to the intensive care unit included demographic variables (age, sex), admission status (emergency vs. elective), admitting clinical department, comorbidities, APACHE II score, and admission laboratory parameters (albumin, alanine aminotransferase, aspartate aminotransferase, lactate dehydrogenase, total bilirubin, sodium, urea, creatinine, estimated glomerular filtration rate, white blood cell count, neutrophil count, prothrombin time, activated partial thromboplastin time, international normalised ratio, and procalcitonin).

### 2.4. Statistical Analysis

The sample size was determined by the number of eligible patients during the pre-specified study period. With 84 mortality events in 500 patients and five covariates retained in the final multivariable model, the events-per-variable ratio was 16.8, exceeding the conventional threshold of 10 events per variable for logistic regression.

This ratio reflects only the five predictors retained in the final model. Considering the full candidate pool of 14 variables entered into backward elimination, the events-per-variable ratio was lower (84/14 = 6.0), below the conventional threshold of 10. This should be considered when interpreting model stability.

Continuous variables were tested for normality with the Shapiro–Wilk test; all departed from normality (*p* < 0.05), and non-parametric methods were applied throughout. Shapiro–Wilk testing at this sample size (*n* = 500) has high power to detect even trivial deviations from normality. Skewness was therefore also examined for each variable. Braden score and sodium showed only mild skewness (0.26 and 0.68, respectively), while APACHE II, INR, and procalcitonin showed pronounced right skew (1.37, 12.0, and 13.0, respectively). Non-parametric methods were retained for all variables for methodological consistency, regardless of the magnitude of departure from normality for any individual variable. Between-group comparisons used the Mann–Whitney U test for continuous and chi-square (or Fisher’s exact) test for categorical variables. Continuous and ordinal variables are summarised as median (IQR), categorical variables as count and percentage.

The admission Braden score was analysed both as a continuous predictor (per one-point decrease) and as a binary variable using an ROC-derived threshold. Its association with in-hospital mortality was first assessed by univariable logistic regression and ROC analysis, with the optimal cut-off identified by the Youden J index.

A multivariable logistic regression model was then constructed adjusting for significant univariable predictors. APACHE II was deliberately excluded from the multivariable model and the derived risk score, as it requires integration of multiple physiological and laboratory parameters that are not consistently available at the bedside in real time; one of the secondary aims of this study was to develop a tool applicable at the point of care without dependence on APACHE II computation. The impact of this exclusion on the independence of the Braden score was subsequently examined in a sensitivity analysis (Section 3.7). Multicollinearity was assessed with pairwise Spearman rank correlations; no pair of candidate variables exceeded the threshold of |r| > 0.70. Variance inflation factors (VIF) were also calculated for the five variables retained in the multivariable model; all were below 1.1 (range 1.03–1.05), confirming the absence of meaningful multicollinearity. Final variable selection used backward elimination by AIC. Model performance was evaluated by AUC, Nagelkerke and McFadden pseudo-R^2^, and Youden-index-derived probability threshold.

A simplified bedside risk score (range 0–13) was derived by refitting the multivariable model using the same five predictors after dichotomization, and assigning integer weights proportional to the resulting β coefficients. All four dichotomization thresholds (Braden ≤ 12, INR ≥ 1.4, sodium ≥ 145, procalcitonin ≥ 0.5) were derived from Youden J index analysis on this dataset. The INR and procalcitonin cut-offs were rounded to the nearest clinically conventional value. Patients were stratified into four risk categories (low, intermediate, high, very high), and discriminative performance was assessed by ROC analysis with 1000-fold bootstrap internal validation. The optimism-corrected bootstrap for the risk score used random seed 42; the calibration bootstrap (Section 3.9) used random seed 123.

All analyses used Python 3.13 (pandas 2.3.3, numpy 2.3.5, statsmodels 0.14.5). Two-sided *p* < 0.05 was considered significant.

## 3. Results

### 3.1. Study Population and Outcomes

**Figure 1:**
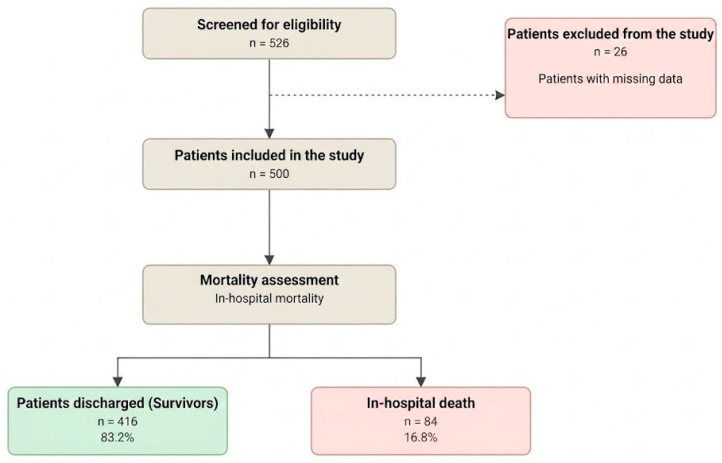
Patient flow diagram. Of 526 patients screened during the study period, 26 were excluded due to incomplete medical records, yielding an analytic cohort of 500 patients admitted to the ICU following a ground-level fall; 84 (16.8%) died in-hospital and 416 (83.2%) survived to discharge.

A total of 500 patients were included in the study (Figure 1). While 84 patients (16.8%) died in hospital, 416 patients (83.2%) were discharged. The cohort consisted of a geriatric group. The median age was 83 (IQR, 74–89; mean 79.9, SD 13.1) and 318 patients (63.6%) were female. The patients had injuries treated by the orthopaedics (*n* = 427, 85.4%) and neurosurgery (*n* = 50, 10.0%) departments. The most common comorbidities were hypertension (*n* = 351, 70.2%), diabetes mellitus (*n* = 162, 32.4%), coronary artery disease (*n* = 124, 24.8%) and dementia (*n* = 103, 20.6%). Hip and long bone fracture surgery accounted for the majority of surgical interventions, whilst 86 patients (17.1%) did not undergo surgery. The median length of hospital stay was 3 days (IQR, 2–7); the length of stay for those who did not survive was significantly longer than for survivors (8 days versus 3 days, *p* < 0.001).

### 3.2. Braden Admission Score and Clinical Comparisons Between Survivors and Non-Survivors

The total Braden admission score was significantly lower in non-survivors than in survivors (median 12 [IQR 11–13] versus 13 [IQR 12–14]; *p* < 0.001). The median APACHE II score on admission was almost twice as high in non-survivors (22 [IQR 15–30] versus 13 [IQR 10–19]; *p* < 0.001), and the proportion of patients admitted directly from the A&E department was significantly higher in the non-survivor group (50.0% versus 19.7%; *p* < 0.001). No significant difference in mortality was observed for age and sex (*p* = 0.841 and *p* = 0.221, respectively). Baseline characteristics classified according to mortality outcome are summarised in Table 1.

### 3.3. Laboratory Findings on Admission

In those who did not survive, the median INR, prothrombin time, activated partial thromboplastin time, urea, creatinine, sodium, total bilirubin, lactate dehydrogenase, aspartate and alanine aminotransferase, white blood cell count, neutrophil count and procalcitonin levels were higher (all *p* ≤ 0.024), whilst albumin levels were lower (32.9 vs. 33.8 g/L; *p* = 0.012) and estimated glomerular filtration rate was lower (59.1 vs. 73.5 mL/min/1.73 m^2^; *p* = 0.008) (Table 2).

### 3.4. Univariable Association Between Admission Braden Score and Mortality

In univariable logistic regression, each one-point decrease in the admission Braden score was associated with a 37.9% increase in the odds of in-hospital mortality (OR 0.725 per one-point increase, 95% CI 0.631–0.833, *p* < 0.001). The discrimination of the admission Braden score for in-hospital mortality, assessed by ROC analysis, yielded an area under the curve (AUC) of 0.676. Optimal sensitivity and specificity, identified by the Youden J index, were observed at a threshold of Braden ≤ 12 (sensitivity 65.5%, specificity 65.9%, positive predictive value 27.9%, negative predictive value 90.4%). At more conservative thresholds, sensitivity rose at the cost of specificity (e.g., Braden ≤ 14: sensitivity 88.1%, specificity 15.4%); at more stringent thresholds, specificity improved (e.g., Braden ≤ 10: sensitivity 21.4%, specificity 94.7%) (Supplementary Table S1).

A total of 20 admission variables reached statistical significance in univariable analysis. Apart from the Braden score, the strongest associations were observed for INR (OR 8.47, 95% CI 3.74–19.35), emergency admission (OR 4.07, 95% CI 2.49–6.66), neurosurgical service admission (OR 3.63, 95% CI 1.94–6.81), procalcitonin (OR 1.03, 95% CI 1.01–1.06), and APACHE II score (OR 1.10, 95% CI 1.07–1.13). Age, sex, and individual comorbidities did not reach significance in univariable analysis (all *p* > 0.05).

### 3.5. Multivariable Analysis: Independent Predictors of Mortality

In the multivariable logistic regression model, the admission Braden score remained an independent predictor of in-hospital mortality after adjustment for clinical and laboratory variables routinely available at ICU admission (adjusted OR 0.821 per one-point increase, 95% CI 0.706–0.954, *p* = 0.010). Four additional variables were retained as independent predictors: emergency admission, INR, serum sodium and procalcitonin; (Table 3). The model demonstrated good discrimination (AUC 0.801, Nagelkerke R^2^ 0.306, McFadden R^2^ 0.222). At the optimal probability threshold identified by the Youden index (predicted probability ≥ 0.240), the sensitivity for in-hospital mortality was 61.9% and the specificity was 88.7%.

### 3.6. Derivation and Performance of the Bedside Clinical Risk Score

A five-component, integer-weighted clinical risk score was derived by refitting the multivariable model using the same five predictors after dichotomization (Table 4). Emergency admission, INR, and procalcitonin each received three points; Braden ≤ 12 and sodium ≥ 145 mEq/L each received two points. The total score ranged from 0 to 13.

When patients were stratified into four risk categories, determined empirically from the observed mortality gradient, in-hospital mortality increased monotonically across categories: 5.0% in the low-risk group (score 0–3; *n* = 321, 64.2%), 22.2% in the intermediate-risk group (score 4–6; *n* = 99, 19.8%), 41.3% in the high-risk group (score 7–8; *n* = 46, 9.2%), and 79.4% in the very-high-risk group (score 9–13; *n* = 34, 6.8%)—a 16-fold gradient in observed mortality from the lowest to the highest stratum. Full model coefficients, standard errors, odds ratios, confidence intervals, and the exact point-assignment rule are provided in Supplementary Table S2.

**Figure 2:**
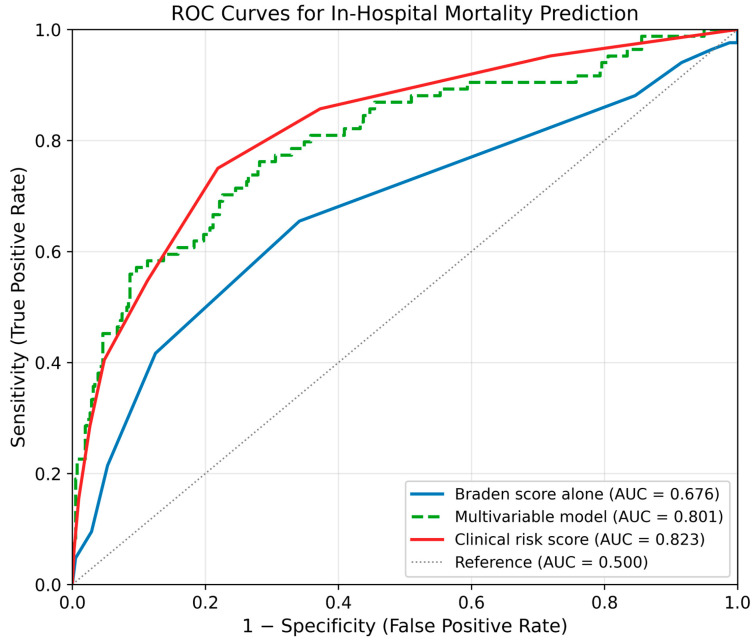
ROC curves comparing discriminative performance for in-hospital mortality: the admission Braden score alone (AUC 0.676), the full multivariable model (AUC 0.801), and the five-component bedside clinical risk score (AUC 0.833).

The simplified clinical score retained the discriminative performance of the full multivariable model: the AUC was 0.833, with a bootstrap-corrected AUC of 0.833 (95% CI 0.780–0.881, 1000 resamples). The discriminative performance of the score exceeded that of the admission Braden score alone (AUC 0.833 vs. 0.676; ΔAUC = 0.157) and was comparable to that of the full multivariable model (ΔAUC = 0.032). A side-by-side comparison of the three models is shown in Figure 2. DeLong testing confirmed that the clinical risk score significantly outperformed the Braden score alone (ΔAUC = 0.157, *p* < 0.0001) and the full multivariable model without the score’s dichotomization (ΔAUC = 0.125, *p* < 0.001 for Braden alone vs. multivariable model). The clinical score did not differ significantly from the full multivariable model (ΔAUC = 0.032, *p* = 0.086), indicating that dichotomization for bedside use did not come at a meaningful cost to discrimination.

### 3.7. Sensitivity Analysis: Adjustment for APACHE II Score

A sensitivity analysis was performed to test whether the admission Braden score remained associated with in-hospital mortality after adjustment for overall illness severity (APACHE II), which had been excluded from the primary model for the reasons described in Section 2.

Four logistic regression models were compared. These were: (i) APACHE II alone; (ii) APACHE II combined with the Braden score; (iii) the original five-variable model (Braden score, emergency admission, INR, sodium, procalcitonin); and (iv) the five-variable model with APACHE II added.

APACHE II alone showed good discrimination for in-hospital mortality (AUC 0.745). Addition of the Braden score to APACHE II did not significantly improve discrimination (AUC 0.760; ΔAUC = 0.015, DeLong *p* = 0.486). However, the Braden score remained independently associated with mortality in this two-variable model (adjusted OR 0.766, 95% CI 0.663–0.884, *p* < 0.001).

APACHE II was then added to the five-variable model. Discrimination improved from an AUC of 0.801 to 0.839 (ΔAUC = 0.038, DeLong *p* = 0.025). In this expanded model, the admission Braden score remained an independent predictor of in-hospital mortality (adjusted OR 0.852, 95% CI 0.730–0.994, *p* = 0.042). Emergency admission (OR 3.256, 95% CI 1.816–5.838, *p* < 0.001), INR (OR 3.778, 95% CI 1.586–9.001, *p* = 0.003), sodium (OR 1.096, 95% CI 1.035–1.160, *p* = 0.002), and APACHE II itself (OR 1.077, 95% CI 1.045–1.110, *p* < 0.001) also remained significant. Procalcitonin was attenuated to non-significance in this model (OR 1.022, 95% CI 0.999–1.046, *p* = 0.067).

### 3.8. Admitting Department, Operative Category, and Mechanical Ventilation

Mortality varied significantly by admitting department. Patients admitted neurosurgery had markedly higher mortality (38.0%) than the rest of the cohort (*p* < 0.001). Orthopaedic admissions had significantly lower mortality (14.0%) than non-orthopaedic admissions (*p* < 0.001). The small neurology and “other” subgroups did not reach statistical significance individually, reflecting limited sample size rather than an absence of clinical risk.

Operative category was also associated with mortality. Patients who did not undergo surgery had significantly higher mortality (42.0%, *p* < 0.0001) than those undergoing hip/joint arthroplasty (10.6%, *p* = 0.004) or long-bone fracture fixation (9.9%, *p* = 0.002). The small “other/vascular procedure” subgroup also showed elevated mortality (38.9%, *p* = 0.026).

Mechanical ventilation showed near-complete separation with in-hospital mortality (97.6% among ventilated patients vs. 0.7% among non-ventilated, *p* < 0.0001). Given this near-perfect association, mechanical ventilation was considered a proximate marker of terminal clinical deterioration rather than an independent risk factor, and was therefore not entered into the multivariable model.

### 3.9. Model Calibration

Calibration of the multivariable model was assessed. The apparent Brier score was 0.105. An optimism-corrected calibration slope was estimated using 1000 bootstrap resamples. This was 0.917 (95% CI 0.661–1.233), close to the ideal value of 1.0. The Hosmer–Lemeshow test showed no evidence of poor fit (χ^2^ = 8.29, df = 8, *p* = 0.406).

Calibration was also assessed for the final simplified integer score itself, rather than the underlying continuous model alone. The optimism-corrected calibration slope was 1.006 (95% CI 0.805–1.243), close to the ideal value of 1.0. The bootstrap-validated Brier score was 0.100. Observed mortality with 95% confidence intervals (Wilson method) by risk category was: Low, 5.0% (95% CI 3.1–7.9%); Intermediate, 22.2% (95% CI 15.2–31.4%); High, 41.3% (95% CI 28.3–55.7%); Very high, 79.4% (95% CI 63.2–89.7%). This bootstrap procedure validates the final, fixed score; it does not re-derive variable or cut-off selection within each resample, which may leave a modest additional source of optimism unaccounted for.

## 4. Discussion

In this single-centre retrospective cohort, a low Braden scale score at admission was associated with higher in-hospital mortality. After multivariable adjustment, a one-point decrease increased the odds of death by approximately 21.8%. Based on this finding, we derived a five-component bedside risk score combining the Braden score with four routinely obtained admission variables. The score yielded a bootstrap-adjusted AUC of 0.833 (95% CI 0.780–0.881) and classified patients across a 16-fold mortality gradient. To our knowledge, this appears to be the first study to evaluate the prognostic value of the admission Braden score in an ICU population defined specifically by ground-level falls.

The Braden scale was developed for pressure-injury risk, not as a formal frailty instrument. Some subscales—mobility, nutrition, sensory perception—conceptually overlap with frailty domains, and prior studies have proposed it as a bedside proxy for physiological reserve [12]. This overlap remains conceptual, as no validated frailty instrument was used in our cohort.

Ground-level falls are generally low-energy trauma, but they can still cause life-threatening injuries such as intracranial haemorrhage, pelvic fracture, or post-operative complications after hip fracture surgery, particularly with reduced physiological reserve or anticoagulant use.

The Braden score therefore offers a practical bedside indicator of physiological reserve, though it cannot separate premorbid frailty from acute injury severity.

This prognostic signal is not unique to our setting. Brueske et al. found that lower Braden subscale scores at admission independently predicted mortality across 11,954 cardiac ICU patients, and Wang et al. reported AUCs of 0.77–0.82 for short-term mortality prediction in 2355 patients with traumatic brain injury [12,13]. Comparable associations have since been described in ischaemic stroke, non-traumatic subarachnoid haemorrhage, sepsis, dementia, and multiple organ dysfunction syndrome [14,15,16,17,18]. Against this backdrop, the AUC of 0.676 observed for the admission Braden score in our cohort is modest but consistent—and, crucially, it was achieved with a single variable recorded during routine nursing assessment.

Emergency department admission was among the strongest independent predictors of mortality in the multivariate model (adjusted OR 3.277). In this patient group, emergency presentation reflects altered consciousness, haemodynamic instability, or active bleeding in anticoagulated patients—each a marker of acute physiological deterioration [19]. Scharringa et al. demonstrated in a multicentre cohort that unplanned admission independently predicted mortality in severely injured geriatric patients [4]. In our cohort, emergency admission was twice as frequent among non-survivors as survivors (50.0% vs. 19.7%; *p* < 0.001).

Coagulopathy in geriatric trauma is multifactorial: chronic liver disease, vitamin K deficiency, age-related decline in clotting factor synthesis, and oral anticoagulant use frequently coexist [20]. INR carried the highest odds ratio in univariable analysis (OR 8.47) and remained a strong independent predictor after adjustment (OR 5.535). The median INR was higher in non-survivors than survivors (1.4 vs. 1.2; *p* < 0.001). This elevation also reflects declining hepatic synthetic capacity and eroded organ reserve [21]. Co-occurrence of elevated INR and intracranial haemorrhage represents a particularly lethal combination—hence the highest weighting in the score (3 points).

Each 1 mEq/L increase in serum sodium increased the likelihood of mortality by 9.6% (adjusted OR 1.096), with median sodium higher in non-survivors than survivors (142 vs. 141 mEq/L; *p* < 0.001). Hypernatraemia in ICU patients may result from fluid restriction, inadequate enteral intake, a hypermetabolic state, or osmotic therapies. In geriatric patients it may initially go unnoticed [22]. Multiple large cohort studies have shown that in-hospital hypernatraemia independently increases mortality in critically ill patients [23]. On admission, it should be read as both a marker of acute physiological impairment and of pre-existing frailty.

All five components can be calculated at the bedside at the time of admission, enabling early mortality prediction. Observed mortality ranged from 5.0% in the lowest risk category to 79.4% in the highest—a gradient that directly informs treatment goals and care planning. The combined score outperformed the Braden score alone (AUC 0.833 vs. 0.676; ΔAUC = 0.157), demonstrating that routine laboratory data add meaningful prognostic value beyond bedside assessment alone.

This study has several strengths. The cohort spans more than four years and the event-to-variable ratio was 16.8 for the final retained predictors, although lower (6.0) across the full candidate pool considered during variable selection (see Section 2). Internal validation was performed by 1000-fold bootstrap resampling. The main limitation is that this was a single-centre, retrospective study; external validation is required before clinical implementation. The retrospective nature also precluded assessment of pre-fall functional status, polypharmacy, and detailed anticoagulant use. Baseline characteristics of the 26 excluded patients could not be compared with the analytic cohort, as their records were incomplete precisely because of the missing data that led to exclusion. The extent and direction of any resulting selection bias could therefore not be formally assessed, though the excluded proportion was modest (4.9% of screened patients). As the dichotomization thresholds for the bedside score were derived from this same dataset, they represent an internal, data-driven derivation rather than an externally validated one; external validation of these cut-offs in an independent cohort is needed before wider clinical use. The bedside score was developed through several sequential steps. These include univariable screening, backward elimination, data-driven cut-off selection, dichotomization, and empirical risk-category formation. Each step carries some risk of overfitting. The score should therefore be considered exploratory and internally derived. It is not yet a validated, ready-to-use clinical instrument. Inter-rater reliability of Braden scoring across nursing staff was not formally assessed in this retrospective design; while institutional nursing protocols standardise training on the scale, this should be considered a limitation. Finally, long-term outcomes—90-day or one-year mortality, functional recovery, discharge status—were not examined. A standardised trauma severity score (ISS/AIS) and detailed fracture classification could not be derived retrospectively. Coagulation-related variables—specific anticoagulant or antiplatelet agent, dose, and reversal treatment—were also not systematically available. This limits our ability to characterise injury severity in detail. As a result, the admission Braden score may partly reflect acute injury severity, neurological impairment, immobilisation, or treatment pathway. It may not purely reflect premorbid frailty or physiological reserve. This distinction could not be resolved in the present design. The precise interval between hospital admission and ICU admission could not be determined. Nor could the interval between ICU admission and Braden scoring. Pre- versus post-operative timing was also not available for individual cases. The admission Braden score may therefore be influenced by anaesthesia, sedation, intubation, pain, delirium, or postoperative immobilisation. It should not be interpreted as a clean marker of baseline, premorbid frailty. These represent directions for future prospective work. The retrospective database did not capture the time interval between hospital admission and ICU admission, the interval between ICU admission and Braden scoring, or whether scoring occurred before or after surgery. The admission Braden score may therefore partly reflect acute peri-injury or peri-operative physiological perturbation (e.g., sedation, pain, immobilisation) rather than premorbid functional reserve alone, and this distinction could not be disentangled in the present design.

The admission Braden score was associated with in-hospital mortality following ground-level fall. When combined with emergency admission, INR, serum sodium, and procalcitonin, discriminative performance improved substantially (AUC 0.833). All five components are routinely available within the first hours of ICU admission. The score has only undergone internal validation in this single-centre cohort. Multicentre prospective studies are needed for external validation before clinical implementation.

## Figures and Tables

**Table 1:** Baseline demographic and clinical characteristics of the cohort, stratified by in-hospital mortality (*n* = 500).

Variable	Survivors (*n* = 416)	Non-Survivors (*n* = 84)	*p* Value
Age, years, median (IQR)	83 (74–89)	83 (75–89)	0.841 (*)
Female sex, *n* (%)	270 (64.9)	48 (57.1)	0.221 (**)
Emergency admission, *n* (%)	82 (19.7)	42 (50.0)	<0.001 (**)
ICU length of stay, days, median (IQR)	3 (2–7)	8 (4–21)	<0.001 (*)
APACHE II score, median (IQR)	13 (10–19)	22 (15–30)	<0.001 (*)
Admission Braden total score, median (IQR)	13 (12–14)	12 (11–13)	<0.001 (*)

IQR: interquartile range, (*) Mann–Whitney U test; (**) chi-square test. APACHE II = Acute Physiology and Chronic Health Evaluation II.

**Table 2:** Admission laboratory parameters by in-hospital mortality status (*n* = 500).

Variable	Survivors (*n* = 416)	Non-Survivors (*n* = 84)	*p* Value
Albumin, g/L	33.8 (31.6–36.9)	32.9 (28.3–36.3)	0.012
INR	1.2 (1.1–1.3)	1.4 (1.2–1.6)	<0.001
Prothrombin time, s	13.3 (12.5–14.4)	15.2 (13.2–17.9)	<0.001
aPTT, s	25.4 (22.9–28.6)	29.8 (24.8–34.6)	<0.001
Sodium, mEq/L	141 (139–143)	142 (139.8–147)	<0.001
Urea, mg/dL	59.9 (44.2–81.9)	89.3 (59.7–118.4)	<0.001
Creatinine, mg/dL	1.0 (0.7–1.4)	1.5 (1.0–2.3)	<0.001
eGFR, mL/min/1.73 m^2^	73.5 (48.9–86.7)	59.1 (39.8–81.8)	0.008
Total bilirubin, mg/dL	0.9 (0.6–1.2)	1.2 (0.7–1.7)	<0.001
AST, U/L	34 (25–51)	62 (31–103)	<0.001
ALT, U/L	20 (15–27)	27 (17–75)	<0.001
LDH, U/L	325 (290–365)	380 (300–517)	<0.001
Procalcitonin, ng/mL	0.3 (0.1–0.6)	0.8 (0.4–3.5)	<0.001
WBC, ×10^3^/μL	11.9 (10.0–15.1)	13.6 (9.9–17.1)	0.024
Neutrophil count, ×10^3^/μL	10.4 (8.2–13.3)	11.7 (8.5–15.5)	0.012

Values are median (IQR). Comparisons performed with the Mann–Whitney U test. INR = international normalised ratio; aPTT = activated partial thromboplastin time; eGFR = estimated glomerular filtration rate; AST = aspartate aminotransferase; ALT = alanine aminotransferase; LDH = lactate dehydrogenase; WBC = white blood cell count.

**Table 3:** Multivariable logistic regression model for in-hospital mortality (*n* = 500).

Variable	Adjusted OR	95% CI	*p* Value
Braden total score (per 1-point increase)	0.821	0.706–0.954	0.010
Emergency admission	3.277	1.878–5.718	<0.001
INR (per 1-unit increase)	5.535	2.365–12.952	<0.001
Sodium, mEq/L (per 1-unit increase)	1.096	1.038–1.157	<0.001
Procalcitonin, ng/mL (per 1-unit increase)	1.025	1.003–1.047	0.024

Variables retained after backward elimination (Akaike information criterion). OR = odds ratio; CI = confidence interval; INR = international normalised ratio. Model performance: AUC = 0.801, Nagelkerke R^2^ = 0.306, McFadden R^2^ = 0.222, *n* = 500, events = 84, events-per-variable = 16.8. Overall model likelihood-ratio *p* < 0.001; individual *p*-values from Wald tests.

**Table 4:** Components, point weights, and risk-stratification performance of the bedside clinical risk score.

(a) Score components and point weights				
**Variable and Cut-Off**			**Points**	
INR ≥ 1.4			+3	
Emergency admission			+3	
Braden total score ≤ 12			+2	
Sodium ≥ 145 mEq/L			+2	
Procalcitonin ≥ 0.5 ng/mL			+3	
**Maximum total**			**13**	
(b) Risk categories and observed in-hospital mortality				
**Risk category**	**Total score**	***n* (%)**	**Observed mortality**	**%95 CI**
Low	0–3	321 (64.2%)	16 (5.0%)	3.1–7.9%
Intermediate	4–6	99 (19.8%)	22 (22.2%)	15.2–31.4%
High	7–8	46 (9.2%)	19 (41.3%)	28.3–55.7%
Very high	9–13	34 (6.8%)	27 (79.4%)	63.2–89.7%
(c) Discriminative performance compared with the Braden score alone and the full multivariable model				
**Performance metric**		**Braden alone**	**Multivariable model**	**Clinical risk score**
AUC		0.676	0.801	0.833
Bootstrap-corrected AUC (95% CI)		—	—	0.833 (0.780–0.881)
Sensitivity (optimal cut-off)		65.5%	61.9%	81.0%
Specificity (optimal cut-off)		65.9%	88.7%	73.3%
Optimal cut-off		Braden ≤ 12	Probability ≥ 0.24	Score ≥ 4

Internal validation by 1000-fold non-parametric bootstrap resampling; optimal cut-offs identified by the Youden J index. Pairwise AUC comparisons used the DeLong test (see Section 3.6 for exact *p*-values). AUC = area under the receiver operating characteristic curve; CI = confidence interval.

## Data Availability

The data presented in this study are available on request from the corresponding author due to ethical reasons.

## Supplementary Materials

The following supporting information can be downloaded at: https://www.mdpi.com/article/10.3390/diagnostics16152309/s1, Table S1: Sensitivity, specificity, positive predictive value, and negative predictive value of the Braden score at adjacent cut-off values for in-hospital mortality prediction. Sensitivity, specificity, positive predictive value (PPV), and negative predictive value (NPV) of the admission Braden scale score at adjacent cut-off values for the prediction of in-hospital mortality Table S2: Binary-predictor logistic regression model (coefficients, standard errors, odds ratios, 95% confidence intervals, and the exact point-assignment rule) underlying the derivation of the bedside clinical risk score.
