# Supplementary material for: Admission Braden Scale Score Is Associated with In-Hospital Mortality in ICU Patients Admitted After a Same-Level Fall: A Retrospective Cohort Study
Source: Diagnostics (Basel). 2026 Jul 23;16(15):2309. doi: 10.3390/diagnostics16152309 (PMC13463849; doi:10.3390/diagnostics16152309)
Supplement: Supplementary file 1 [file diagnostics-16-02309-s001.zip › diagnostics-4390855-supplementary.pdf]

Supplementary Table S1. Sensitivity, specificity, positive predictive value (PPV), and negative predictive value (NPV) of the admission Braden Scale score at adjacent cut-off values for the prediction of in-hospital mortality (*n* = 500).

Braden Threshold	Sensitivity (%)	Specificity (%)	PPV (%)	NPV (%)	N at Risk	Note
≤ 8	4.8	99.5	66.7	83.8	6	
≤ 9	9.5	97.1	40.0	84.2	20	
≤ 10	21.4	94.7	45.0	85.7	40	
≤ 11	41.7	87.5	40.2	88.1	87	
**≤ 12**	**65.5**	**65.9**	**27.9**	**90.4**	**197**	**← Optimal**
≤ 13	79.8	33.9	19.6	89.2	342	
≤ 14	88.1	15.4	17.4	86.5	426	
≤ 15	94.0	8.4	17.2	87.5	460	
≤ 16	96.4	3.8	16.8	84.2	481	
≤ 17	97.6	1.2	16.6	71.4	493	

PPV = positive predictive value; NPV = negative predictive value. N at risk = number of patients with Braden score ≤ threshold out of n = 500. The ***optimal cut-off (Braden ≤ 12)*** *was identified by maximising the Youden J index (sensitivity + specificity − 1). Bold rows indicate the optimal threshold.*

Youden J = Sensitivity + Specificity − 1. Maximum J at Braden ≤ 12: J = 0.655 + 0.659 − 1 = 0.314.

Supplementary Table S2

Binary-predictor logistic regression model used to derive the bedside clinical risk score point weights (refit on dichotomized predictors; see manuscript Section 2.4 and Section 3.6).

Variable	β	SE	OR	95% CI	p value
Intercept	−3.753	0.319	0.023	0.013–0.044	<0.0001
Braden total score ≤ 12	1.111	0.291	3.036	1.715–5.376	0.0001
Emergency admission	1.291	0.293	3.636	2.047–6.458	<0.0001

INR ≥ 1.4	1.353	0.316	3.868	2.082–7.189	<0.0001
Sodium ≥ 145 mEq/L	1.121	0.318	3.068	1.644–5.723	0.0004
Procalcitonin ≥ 0.5 ng/mL	1.227	0.294	3.410	1.919–6.062	<0.0001

Model fit: log-likelihood = −165.52; AIC = 343.05; McFadden pseudo-R^2 = 0.269. All five predictors were entered as binary (0/1) variables using the cut-offs listed above.

Point-assignment rule

Integer point weights were assigned as: Points = round(3 × β / β_max), where β_max is the largest coefficient among the five predictors (INR ≥ 1.4, β = 1.353). Applying this rule:

- Braden ≤ 12: round(3 × 1.111 / 1.353) = round(2.46) = 2 points.
- Emergency admission: round(3 × 1.291 / 1.353) = round(2.86) = 3 points.
- INR ≥ 1.4: round(3 × 1.353 / 1.353) = round(3.00) = 3 points.
- Sodium ≥ 145 mEq/L: round(3 × 1.121 / 1.353) = round(2.49) = 2 points.
- Procalcitonin ≥ 0.5 ng/mL: round(3 × 1.227 / 1.353) = round(2.72) = 3 points.

Maximum total score = 13 (2 + 3 + 3 + 2 + 3). This reproduces exactly the point weights reported in Table 4 of the main manuscript.
